# Development of an Audit Tool to Evaluate End of Life Care in the Emergency Department: A Face and Content Validity Study

**DOI:** 10.1111/jep.70041

**Published:** 2025-02-19

**Authors:** Melissa Heufel, Sarah Kourouche, Rebecca Mitchell, Magnolia Cardona, Benjamin Thomas, Wing‐Shan Angela Lo, Marghie Murgo, Debbie Vergan, Kate Curtis

**Affiliations:** ^1^ Susan Wakil School of Nursing and Midwifery, Faculty of Medicine and Health University of Sydney Sydney New South Wales Australia; ^2^ Emergency Department Wollongong Hospital, Illawarra Shoalhaven Local Health District Wollongong New South Wales Australia; ^3^ Australian Institute of Health Innovation Macquarie University Sydney New South Wales Australia; ^4^ School of Psychology, Faculty of Health and Behavioural Sciences The University of Queensland Brisbane Queensland Australia; ^5^ Institute for Evidence Based Healthcare Bond University Gold Coast Queensland Australia; ^6^ Palliative Care Service, Illawarra Shoalhaven Local Health District Warrawong New South Wales Australia; ^7^ Graduate School of Medicine Wollongong New South Wales Australia

**Keywords:** clinical assessment, clinical decisions, communication, palliative care, service evaluation, symptoms and symptom management, terminal care

## Abstract

**Objectives:**

Emergency Departments (ED) are increasingly caring for patients with acute, chronic and terminal conditions requiring End of Life Care (EOLC). There is no published and validated tool available to evaluate EOLC delivery of patients dying in the ED. This study describes the face and content validity testing process to develop, refine and test a new and unique audit tool to evaluate EOLC in the ED.

**Methods:**

The face and content validation process used a three‐round modified‐Delphi technique. We consulted 11 experts to assess the proposed 89 items. Face validity explored the overall question of appropriateness and relevance; and content validity examined relevance ratings using the Content Validity Index (CVI) 4‐point Likert scale in two rounds. Iterative assessment of ratings led to inclusion (CVI > 0.78), revision (CVI 0.65 to < 0.78) or exclusion (CVI < 0.65) of items from the tool.

**Results:**

Of the initial 89 items, 66 were included (CVI > 0.78), 16 items revised (scores 0.65 to < 0.78), seven were removed (scores < 0.65) and two new items suggested. Items covered the constructs patient characteristics, circumstances of death, ED performance, communication and care planning, recognition of dying, care delivery, and needs of families and carers. Scale CVI achieved 0.90. The consolidated list of 81 items achieved acceptable face validity and excellent content validity.

**Conclusion:**

Face and content validity of the ED EOLC audit tool achieved acceptable item‐CVI scores and an excellent scale‐CVI score. We recommend external validation of its components in real‐life settings to monitor and set locally relevant clinical practice benchmarks.

## 1 Introduction

Emergency departments (ED) often conduct invasive and aggressive treatments to sustain life. However, these life‐saving measures are not always appropriate or reflective of patient wishes [1, 2]. The provision of End Of Life Care (EOLC) requires a complex approach that meets the physical, emotional, spiritual and psychosocial needs of patients and their families and carers [3]. It is recognised that the ED is not the most suitable environment for the delivery of EOLC [4, 5]. However, due to an ageing population with growing numbers of chronic, progressive health conditions hospitals are seeing an increasing number of patients presenting to the ED requiring EOLC, and these numbers are expected to rise [6, 7].

Standards pertaining to EOLC exist in Australia and internationally [8, 9, 10, 11, 12], along with processes that enable the review of care [13, 14, 15]. In Australia, the National Safety and Quality in Health Care Standards, developed by the Australian Commission on Safety and Quality in Health Care (ACSQHC) are used to guide the provision of care in healthcare organisations, yet the audit toolkit developed for reviewing EOLC specifically excludes the ED from its criteria for use [16]. Clinical audits are frequently used in healthcare to review clinical care delivery, and to identify areas for improvement [17, 18].

Our 2023 scoping review of 58 articles, including both published and grey literature that included government reports or policy documents from relevant government or industry bodies, did not identify an audit tool to measure EOLC delivery which included measures pertinent to the ED environment. In fact, many existing audit tools excluded the ED [19]. The exclusion of this vulnerable subset of patient prevents the identification and quantification of opportunities to improve the experience of patients at the EOL in the ED. As part of our 2023 review, we identified 10 overarching categories of information collected in the audit tools; patient characteristics, physical components of care, communication and care planning, the needs of families and carers, the identification of dying, spiritual, cultural and religious needs, emotional needs, the environment that death took place. We integrated these results with national quality standards, existing ED process measures and emerging literature pertaining to EOL screening tools. This resulted in seven constructs of care that informed the development of a draft 89 item audit tool to audit EOL care in the ED. A full description of the development of the tool is described elsewhere [19].

The intention of clinical audit is to examine current practices and compare these with established clinical standards. This facilitates the identification of gaps in care delivery so that improvements can be planned and implemented to ensure care is delivered at a level that meets quality standards. To do so, a reliable data collection tool is required [20] and instruments that are suitable and relevant to the chosen construct and population under study are essential [21]. Before use, it is essential that a new tool undergoes rigorous testing to ensure validation for clinical use and avoid low‐value data collection that does not enrich the clinical audit process and subsequently practice [20]. Validity assessment ensures a data collection tool contains items pertinent to the domain being explored [20]. Validity is ‘the ability of an instrument to measure the attributes of the construct under study’ [22]. There are various types of validity, though face and content validity are considered critical early steps [23]. This study aims to describe the face and content validity testing process taken to develop, refine and test a new and unique audit tool to evaluate EOLC in the ED.

## 2 Methods

### 2.1 Design and Sample

A three‐round modified‐Delphi study was undertaken to obtain expert opinion on the content and overall appropriateness of the audit tool. Round one examined the face validity of the ED EOLC audit tool and round two examined content validity. The study was conducted in Australia, with panellists from various geographical areas and health districts from across New South Wales and Queensland, carried out from September 2022 to May 2023. A purposive sample of experts comprising clinical specialists in emergency medicine, palliative care, primary care, social work and theme‐specific researchers were invited to participate. Purposive sampling was selected as both face and content validity testing requires the selection of relevant experts on the subject [23, 24]. In an attempt to counter potential response bias, given that subjective assessments must be made for both face and content validity, we ensured the inclusion of experts from different locations, health districts and sectors. For rounds two and three, content validity testing used the Content Validity Index (CVI) and it is recommended that between 8 and 12 experts [23] are involved in the rating, as a greater number of experts (i.e., at least 10) diminishes the probability of chance agreement using the CVI.

### 2.2 Face Validity

Face validity is a subjective assessment of whether a tool appears to measure the construct of interest [25]. In round one, panellists were provided a Microsoft Word document with a copy of the draft audit tool and were asked to provide comment on the relevance to EOLC in the ED, ambiguity and overall appropriateness of the tool and were asked to identify any unnecessary or missing items. Panellists could also add items they felt were missing.

### 2.3 Content Validity

Content validity is considered the extent to which the items individually, and as a whole, represent the construct of interest, and the CVI is the most widely used method of testing content validity [23, 26]. The CVI is the quantification of item relevance based on expert ratings for each item [23, 26, 27]. For this study, the recommendations for CVI testing and analysis outlined by Polit et al. [23] and Yusoff [27] were followed. Two rounds of CVI testing were conducted for this study as is suggested by Polit et al. [23]. The first round comprised the initial testing of the 89 items that were included in the tool and the second round was undertaken to reassess those items requiring revision and any new items suggested in the first round. For both rounds 1 and 2, REDCap (a secure web‐based application for data collection and storage) was used to formulate an online content validation form, which was sent to the expert panellists with instructions on completing the form (a copy of the two forms can be found as Supporting Information).

Calculating item (I‐CVI) and scale level CVI (S‐CVI) involves asking experts to rate the relevance of each item on a 4‐point likert scale, from 1—*not relevant*, 2—*somewhat relevant*, 3—*quite relevant*, to 4—*highly relevant*. The I‐CVI score is calculated first and once inclusion, removal or revision of items is complete the S‐CVI can be calculated. Panellists were encouraged to provide additional written comments. Reminder emails were sent to panellists at the end of the first and second week. Microsoft Excel was used for analysis.

### 2.4 Data Management and Analysis

Data were exported to Microsoft Excel for analysis. Relevance ratings were recoded as 1 (ratings of 3 or 4) or 0 (ratings of 1 or 2). Each item is assigned an I‐CVI score by adding the number of experts in agreement and dividing by the total number of experts. An I‐CVI value was considered excellent if a rating of at least 0.78 is achieved [23], these items were kept as is and included in the tool, items receiving less than 0.65 were deleted and between 0.65 and < 0.78 were revised.

As the S‐CVI/Universal Agreement can be considered to be overly stringent and harder to achieve with a greater number of experts and ignores the risk of chance agreement [23], we calculated the S‐CVI as the S‐CVI/Average (Ave). The S‐CVI/Ave was calculated using the average of all I‐CVI scores across all items. The recommended value for excellent content validity is an S‐CVI/Ave of ≥ 0.90. By using the S‐CVI/Ave and at least 10 experts we removed the need to also calculate a modified kappa score [23].

## 3 Results

Eleven experts were purposively selected for invitation to participate in the study based on expertise and experience. All 11 agreed to participate, however one expert withdrew from the study for the second round of CVI testing as they had left their clinical position and travelled internationally. The average years of experience in their field was 13 years (range 7–31 years), many of the experts possessed both clinical and research expertise (Table 1).

**Table 1:** Panellist characteristics.

	*n*	%
Educational level		
Bachelor's degree	1	9
Graduate certificate	1	9
Master's degree	6	55
Doctorate degree	3	27
Area of expertise		
Emergency medicine/critical care	4	36
General practice	1	9
Gerontology	1	9
Health outcomes	1	9
Palliative care/end of life	3	27
Social work	1	9
Years of experience		
< 10 years	1	9
10–15 years	5	45
15–20 years	2	18
> 20 years	3	27

### 3.1 Face Validity

All 11 panellists returned feedback on all items in the proposed audit tool within the 2‐week period from September to October 2022, a reminder email was sent to panellists after the end of the first week. Feedback resulted in the addition of eight items and a revision of the wording of four items (Table 2). For example, one panellist suggested ‘should there be a category for triage category change, when situation changes? IE if initially triaged as Cat 4 – re‐identified due to deterioration, up category? And check if that was accurate as well?’ (Panellist 3) and another suggested a change in wording to one of the items stating, ‘Where was the patient prior to hospital admission? Should this be Usual residence of the patient?’ (Panellist 2).

**Table 2:** Items changed based on phase one feedback.

Action	Original wording	Revised wording
Section 1: Patient characteristics		
Changed	Where was patient before hospital admission?	Place of usual residence
Added		Is patient known to community palliative care service?
Added		Total hospital length of stay (hours)
Section 2: Circumstances of death		
Added		Were specialist palliative care contacted for advice? If yes, date/time
Section 3: ED performance		
Added		If patient deteriorated before medical officer review, was the triage category appropriately upgraded?
Section 4: Communication and care planning		
Added		If resuscitation plan was revised/changed—Date/time of revision and what changes were made
Changed	At any point was there evidence of conflicting orders that might create confusion about the patient's resuscitation status or the medical treatments that were limited?	At any point was there evidence of conflicting statements that might create confusion about the patient's resuscitation status or the medical treatments that were limited?
Added		Was the patient's usual GP/GP practice contacted for information regarding patient's usual status, current palliative care arrangements and illness trajectory/likelihood of death?
Added		Was the patient's usual GP/GP practice sent a discharge summary following the patient's death?
Section 6: Care delivery		
Changed	Is there documented evidence that anticipatory medication was prescribed for symptoms likely to occur in the last days of life?	Is there documented evidence that anticipatory medication was prescribed appropriately for symptoms likely to occur in the last days of life? (must include one opioid, one sedative and one antisecretory—Of subcutaneous administration)
Added		If yes, date/time
Changed	Is there documented evidence that unnecessary medications were ceased?	Once a decision for EOL care was made were regular medications which may have been thought to be unnecessary ceased?

Abbreviations: EOL = end of life, GP = general practitioner.

There were also recommendations given regarding the structure of available responses to some of the items, for example, one panellist stated ‘I'd recommend you format the data extraction tool with check boxes’ (Panellist 6) and another panellist stated ‘is there opportunity for a “Comments” open‐ended response at the end of the section?’ (Panellist 3). These comments resulted in a change of structure of some of the items in the tool for appropriate sections to have checkboxes or drop‐down responses and the addition of a comments box at the end of each section. After modifications were made to the audit tool following expert review, face validity was considered acceptable, and ready for content validity testing.

### 3.2 Content Validity

For round one of the CVI testing, the REDCap online content validation form was distributed to panellists for a 3‐week period between April and March 2023, reminder emails were sent at the end of both Week 1 and 2. There were 89 items included in the first round of CVI testing. Sixty‐six items received scores greater than 0.78 thus deemed acceptable for inclusion in the tool without any changes. Seven items received scores lower than 0.65 so were removed, and there were 16 items that received scores between 0.65 and 0.78 so required further revision and review. Many of the items which received high scores were directly related to the clinical assessment and care of the patient during the presentation, such as symptom assessment, communication and consideration of patients wishes. Those which received lower scores and likely to be deleted were those that were more directly related to history or processes. For example, the question ‘Was the patient known to community palliative care services’ scored low at 0.64 thus deleted, and comment by one of the panellists to this question says ‘Community palliative care is somewhat relevant but not a be‐all end all, as a lot of death is unpredictable’ (Panellist 9). Of the 16 items for revision, four received scores < 0.65 however comments left by the expert panel suggested there was perhaps misunderstanding or views that required further assessment. For example, in relation to Section 7, bereavement care, one panellist scored it as not relevant however the comment states ‘I think this section is relevant but perhaps if it did not happen, should not be used in the audit to detract from the quality of care offered to the patient’ (Panellist 4). While scoring indicated not relevant the comment suggested otherwise so was included for the second round of CVI testing with further clarification given. A summary table which provides the I‐CVI for each item in round one can be found in the Supporting Information.

The 16 items for revision were reviewed by the research team; there were some minor changes to wording for a minority of items and further definition and clarification provided for the items to be sent to the panellists for review. Two items were added following suggestions made by experts in the first round. A new REDCap online content validation form, containing the items which required rereview, was distributed to panellists for a 3‐week period between April and May 2023, and reminder emails were sent at the end of both Week 1 and 2. There were 10 completed responses to the second round. A summary table which provides the I‐CVI for each item in round 2 can be found in the Supporting Information. Twelve items received a score of ≥ 0.78 and were included. Three items received scores < 0.65 and were removed. There were three items that received scores between 0.65 and 0.78 and were revised by the research team, after considering the comments from panellists and were included. For example, one items comments suggested the item could be ‘hard to define’ (Panellist 7) and for the same item another panellist stated ‘A better format should be a checklist with boxes to tick what was NOT ceased that should have been’ (Panellist 4) so this item was included with modifications, with an I‐CVI of 0.70.

Following the second round of I‐CVI analysis, the I‐CVI results indicated acceptable content validity of the items and the S‐CVI could then be calculated. The S‐CVI/Ave received a score of 0.9. A score of 0.9 is considered excellent for scale level content validity, and thus content validity for the ED EOLC audit tool was achieved.

## 4 Discussion

This practical and evidence‐based approach to determine the face and content validity of the ED EOLC audit tool covered a range of aspects to consider in a quality of EOLC assessment: constructs of patient characteristics, circumstances of death, ED performance, communication and care planning, recognition of dying, care delivery and needs of families and carers. The final EOLC audit tool comprised 81 items and achieved acceptable face validity and excellent content validity. The process resulted in alterations to the tool, from minor wording revisions to removal of some items identified as not relevant by the expert panel. Face and content validity testing was a lengthy and iterative process but invaluable in generating constructive insights into the individual audit items and the audit tool as a whole.

The development of national standards related to end of life care delivery is evolving in many countries [8, 9, 10, 11, 12] and subsequently, the review of end of life care processes [13, 14, 15]. In Australia specifically, where the authors of this study are based, a National Consensus Statement pertaining to essential elements in the delivery of care to patients at the EOL in acute care settings were introduced in 2015 [8] and embedded into National Safety and Quality in Healthcare Standards (NSQHS) in 2017 [16]. These standards have been developed to ensure that healthcare services have a framework to achieve high‐quality EOLC for patients and their families/carers. Audit and review of the delivery of EOLC is an essential action outlined in these standards [16], yet the audit toolkit that has been developed by the ACSQHC excludes the ED in its criteria for use, as do other similar audits internationally [14, 15, 28]. A 2023 scoping review conducted as part of the development of the audit tool detailed in this paper, identified and compared 58 international articles describing audit tools related to reviewing EOLC. As no tool for the ED was found, a detailed comparison was conducted and is reported elsewhere [29].

Currently, with no EOLC audit tool that considers the unique nature of the ED environment and with many existing EOLC audit tools excluding the ED from the audit process, there is a vulnerable subset of patients who are not being considered for care evaluations and potential identification of gaps and improvements that clinical audits can achieve. The considerable steps taken to ensure the relevance and validity of the ED EOLC audit tool will bridge this gap and enable ED staff to undertake clinical audit using a tool that is evidence‐based and not only meets national standards but ensures that achievements and gaps in EOLC delivery are identified, and any opportunities for improvement can be recognised.

It is recognised that there are potential barriers to use of any audit tool in the clinical environment beyond those with which can be accounted for in the development phase. Organisational barriers such as time and resource constraints are among the most reported, as is lack of skilled expertise [30]. Considering barriers that may be faced, considerations to ease the burden need to be explored both in the development and implementation phase. For example, due to the length of the tool, where feasible, automatic extraction of the items from electronic records would speed the audit process for items that do not require subjective clinical judgement and should be incorporated. Equally to exploring barriers, facilitators to clinical audit should be considered. The ED EOLC audit tool was designed for use by clinicians and managers who are involved in the review of patient care, using local capabilities, ownership and supportive organisational culture are identified as facilitators to the success of implementation of clinical audit [30].

Face and content validity is an early and important step in the tool development process, and this study has reported the process to achieve this validity. A copy of the ED EOLC audit tool following face and content validity is provided in the Supporting Information. Future research to refine and develop the ED EOLC audit tool should include reliability testing and consider other strategies that may enhance the suitability and authenticity of the ED EOLC audit tool, such as incorporating feedback from families and patients, as well as expanding testing to ED settings outside of the Australian context.

### 4.1 Limitations

There are several limitations to this study. Face validity and content validity both require subjective judgements which could introduce bias to the assessments of experts and could impact the outcome of items included. To attempt to counter response bias, we ensured the inclusion of experts who were not limited to one location, health district or health sector. For content validity, experts are only able to judge the content validity of what items are included. To minimise this limitation, opportunities were provided during the face validity phase and the first round of content validity testing for the experts to provide additional comments or suggestions, and as such items were added for the wider expert group to evaluate through the content validity process. We acknowledge that the tool may not be applicable to EDs in all health systems.

## 5 Conclusion

Face and content validity of the ED EOLC audit tool achieved acceptable I‐CVI scores and an excellent S‐CVI/Ave score. The ED EOLC audit tool was developed based on a comprehensive review of the literature, and a rigorous validation process. Further research is required to ensure the usability, reliability and acceptability of the tool in the clinical environment.

## Ethics Statement

This study was approved by the Illawarra Shoalhaven Local Health District Low and Negligible Risk (LNR) Research Review Committee (LNR reference number ISLHD/LNR/2022‐200). All panellists were provided with a participant information sheet outlining the background of the study and information about the requirements.

## Consent

Participation in the study was voluntary and completion of the online form was considered consent to participate.

## Conflicts of Interest

The authors declare no conflicts of interest.

## Supporting information

Supporting information.

Supporting information.

Supporting information.

Supporting information.

## Data Availability

The data that supports the findings of this study are available in the Supporting Information of this article.
