# Supplementary material for: Development of an Audit Tool to Evaluate End of Life Care in the Emergency Department: A Face and Content Validity Study
Source: J Eval Clin Pract. 2025 Feb 19;31(1):e70041. doi: 10.1111/jep.70041 (PMC11839938; doi:10.1111/jep.70041)
Supplement: Supplementary file 1 — Supporting information. [file JEP-31-0-s002.pdf]

ED EOL Audit Tool CVI Revision Survey

A A A

Please complete the survey below.

Thank you!

Emergency Department (ED) end of life audit tool: content validity survey

Thank you for your participation in the ED end of life audit tool content validity study. Your previous responses have been reviewed and Content Validity Index score calculated. Seven items were eliminated and 18 require further review / clarification. This clarification is provided in this new survey (which is much shorter than the first one!)

Please rate each individual item / question based on relevance to evaluating the quality of EOL care for patients who present to the ED and die within 48hours.

1. Not relevant

2. Somewhat relevant

3. Quite relevant

4. Highly relevant

If you have feedback around clarity or item wording, please detail this in the corresponding notes box.

Your feedback is important to the development of the ED end of life audit tool. Ultimately, we hope to improve the management of ED patients at their end of life.

If you have any questions related to this study, please contact mbra0343@uni.sydney.edu.au

Item	Clarification	Relevance
Speciality with overall responsibility for the patient's	While this may not directly influence the quality of end of life care, it may assist to identify trends,	Not relevant / Somewhat relevant / Quite relevant / Highly relevant<br>Comments

care at time of death	bariers and target education	
Specific ward / department died	While this may not directly influence the quality of end of life care, it may assist to identify trends, bariers and target education	**Not relevant** **Somewhat relevant** **Quite relevant** **Highly relevant**<br>Comments
Triage information	If assessing whether triage category was appropriate this enables the information to be shown here - can be extracted directly from eMR	**Not relevant** **Somewhat relevant** **Quite relevant** **Highly relevant**<br>Comments
Was triage category appropriate based on triage information / observations?	Will require level of assessment by someone who is trained in triage. The ATS information sheet is also provided with the audit tool. Item used to assess whether patients at the EOL are being triaged appropriately. This is a usual KPI used in the ED. Anecdotally, patients presenting at the EOL may be undertriaged due to existing limitations of care.	**Not relevant** **Somewhat relevant** **Quite relevant** **Highly relevant**<br>Comments
If patient deteriorated before medical	Patients at the EOL can often be allocated low triage categories and may deteriorate or experience distressing symptoms	**Not relevant** **Somewhat relevant** **Quite relevant** **Highly relevant**

officer review, was the triage category appropriately upgraded per the ATS?	whilst in the ED before being seen by a medical officer. This will assess whether appropriate re-triage processes are being followed. Our local EDs also now have an ED CERS escalation process that may be utilised.	Comments
Prior to presentation was there a hospital resuscitation form on file?	Whilst hospital resuscitation plans are often to be made and renewed on each admission, some are valid for longer periods e.g. outpatient haemodialysis patients, they may provide helpful information that outlines patient wishes that can inform decision-making on current presentation	Not relevant / Somewhat relevant / Quite relevant / Highly relevant<br>Comments
Time from presentation to first resus plan	Will enable review of how long into patients stay decisions surrounding advance care planning were documented on hospital forms that guide treatment and care	Not relevant / Somewhat relevant / Quite relevant / Highly relevant<br>Comments
Was the resuscitation plan revised/changed at any time?	Highlight changes in decision-making and treatment plans during stay	Not relevant / Somewhat relevant / Quite relevant / Highly relevant<br>Comments
At any point was there evidence		Not / Somewhat / Quite / Highly

or conflicting statements that might create confusion about the patient's resuscitation status or the medical treatments that were limited?	Review the quality / clarity of advance care planning / resuscitation plan documentation	relevant	relevant	relevant	relevant
		Comments			
Total CriSTAL score	Criteria for Screening and Triaging to Appropriate aLternative care (CriSTAL) is a tool used to predict short-term mortality. While not deemed suitable for prospective use in the ED we consider its use in retrospectively identfying patients at risk and determining whether advance care planning conversations took place for such patients.	Not relevant	Somewhat relevant	Quite relevant	Highly relevant
		Comments			
Was the patient at high risk of dying (CriSTAL score >6) on arrival to the ED?	Criteria for Screening and Triaging to Appropriate aLternative care (CriSTAL) is a tool used to predict short-term mortality. While not deemed suitable for prospective use in the ED we consider its use in retrospectively identfying patients at risk and determining whether advance care planning conversations took place for such patients.	Not relevant	Somewhat relevant	Quite relevant	Highly relevant
		Comments			
Recognition of dying date and	Collecting this data will enable comparisons to other data items that may reflect quality EOL care, e.g. time of recognition	Not relevant	Somewhat relevant	Quite relevant	Highly relevant
		Comments			

time	and time of anticipatory medicaton prescription	
Evidence of patient assessment: Nausea / vomiting	A common symptom experienced at the EOL	Not relevant / Somewhat relevant / Quite relevant / Highly relevant<br>Comments
Evidence of patient assessment: Social / practical	Rename to "Social".<br>The need to provide patients with opportunity to have family / friends around / companionship, might include opportunity for assistance for financial support or support with tasks	Not relevant / Somewhat relevant / Quite relevant / Highly relevant<br>Comments
Date and time of anticipatory medications prescription	Collecting this data will enable comparisons to other data items that may reflect quality EOL care, e.g. time of anticipatory medicaton prescription and death	Not relevant / Somewhat relevant / Quite relevant / Highly relevant<br>Comments
If routine care processes were not ceased once	An opportunity for the auditor to provide further free-text details if the documentation indicates that routine care was not	Not relevant / Somewhat relevant / Quite relevant / Highly relevant<br>Comments

a decision for EOL was made, please provide details	ceased once EOL was identified, e.g. still received regular blood tests or NG feeding	
Number of met calls	Assess resource use in the patient at the end of life	Not relevant / Somewhat relevant / Quite relevant / Highly relevant<br>Comments
Is there evidence that families were provided with written bereavement information?	Rephrase to "Is there evidence that families were provided with bereavement information?"	Not relevant / Somewhat relevant / Quite relevant / Highly relevant<br>Comments

Suggested Additonal Question

Is there evidence that bereavement risk screening was undertaken?	Not relevant / Somewhat relevant / Quite relevant / Highly relevant<br>Comments
	Not relevant / Somewhat relevant / Quite relevant / Highly relevant

Were specialist Palliative Care contacted for advice?	Comments

Submit

Save & Return Later
