# Supplementary material for: Development of an Audit Tool to Evaluate End of Life Care in the Emergency Department: A Face and Content Validity Study
Source: J Eval Clin Pract. 2025 Feb 19;31(1):e70041. doi: 10.1111/jep.70041 (PMC11839938; doi:10.1111/jep.70041)
Supplement: Supplementary file 2 — Supporting information. [file JEP-31-0-s004.pdf]

ED EOL audit tool: Content Validity Survey

A A A

Emergency Department (ED) end of life audit tool: content validity survey

We have drafted a tool to review the quality of end of life care for patients who present to the ED and die in hospital within 48 hours. The tool was developed through a review of 58 international research papers, including grey literature (summary info from the literature review is in the file attached below) and consultation with ED, Palliative Care and other experts.

This project is a collaboration with the University of Sydney, the Principal Investigator Melissa Heufel is a Higher Degree by Research student at the University of Sydney and the research will be conducted to fulfil the requirements of a Master of Philosophy (Nursing Research) degree.

We seek your opinion on the level of relevance for each data point in this end of life care audit tool.

Please rate each individual item / question based on relevance.

1. Not relevant
2. Somewhat relevant
3. Quite relevant
4. Highly relevant

If you have feedback around clarity please detail this in the corresponding notes box.

Your feedback is important to the development of the ED end of life audit tool. Ultimately, we hope to improve the management of ED patients at their end of life.

If you have any questions related to this study, please contact mbra0343@uni.sydney.edu.au

Attachment: Supporting information for ED EOL audit tool content validity survey from literature review .docx (70.2 kB)

If you have not read the participant information, a copy can be accessed here (attached below*).*

Please confirm you have read and understood the participant information provided to you

- ○ Yes
- ○ No

Attachment: Participant Information Statement - CVI study.docx (102 kB)

Survey participant characteristics: This information will be used to help support the validation of the tool content, no identifying personal information will be published.

Please list your qualifications:

Area of expertise:

Please provide a short statement outlining your clinical and/or research expertise:

Years of clinical experience, and, working with patients at end of life:

Section 1: Patient Characteristics

Section 1: Patient Characteristics

Rationale: to describe the population and enable monitoring of factors that may contribute to poor or optimal EOL care

Question	*Potential responses*	*Relevance*			
Basic descriptive characteristics<br><br>(Age, sex, country of birth, religion, indigenous status, place of usual residence)		Not relevant	Somewhat relevant	Quite relevant	Highly relevant
Was the patient known to have a terminal / life-limiting illness (e.g. Cancer, COPD, heart failure, dementia)? If yes, please specify	1, Yes<br><br>2, No	Not relevant	Somewhat relevant	Quite relevant	Highly relevant
How many times was the patient	1, None<br><br>2, 1-2 times		Somewhat	Quite	Highly

admitted to an acute hospital in the 12 months prior to this hospital admission?	3, 3-5 times<br>4, More than 5 times<br>5, Not known	Not relevant	relevant	relevant	relevant
Was the patient known to community palliative care services?	1, Yes<br>2, No<br>3, Unsure	Not relevant	Somewhat relevant	Quite relevant	Highly relevant

Do you have any feedback about any of the items in Section 1: Patient characteristics ?

Section 2: Circumstances of death

Section 2: Circumstances of death

Rationale: to develop chronology of events

Question	*Potential responses*	*Relevance*			
Cause of death	(Free text)	Not relevant	Somewhat relevant	Quite relevant	Highly relevant
Speciality with overall responsibility for the patient's care at time of death	(Free text)	Not relevant	Somewhat relevant	Quite relevant	Highly relevant
Specific ward / department died	(Free text)	Not relevant	Somewhat relevant	Quite relevant	Highly relevant
Was the patient admitted to the ICU at anytime during this admission	1, Yes<br>2, No	Not relevant	Somewhat relevant	Quite relevant	Highly relevant

Do you have any feedback about any of the items in Section 2: Circumstances of death?

Section 3: ED performance

Section 3: ED performance

Rationale: to compare patient journey to ED performance measures. The ED setting differs from the inpatient environment, so requires focus on measures unique to ED.

Question	*Potential responses*	*Relevance*			
Mode of arrival	1, State ambulance vehicle<br>2, Community/public transport<br>3, Private vehicle<br>4, Helicopter rescue service<br>5, Air ambulance service<br>6, Internal ambulance / transport<br>7, Police / correctional service vehicles<br>8, Other e.g. undertakers {arrival_mode_other}<br>9, No transport (walked in)<br>10, Retrieval	**Not relevant** ◯	**Somewhat relevant** ◯	**Quite relevant** ◯	**Highly relevant** ◯

	(including NETS)<br>11, Internal bed / wheelchair				
Presenting complaint	(free text)	Not relevant	Somewhat relevant	Quite relevant	Highly relevant
Triage category	1, 1<br>2, 2<br>3, 3<br>4, 4<br>5, 5	Not relevant	Somewhat relevant	Quite relevant	Highly relevant
Triage information	(free text)	Not relevant	Somewhat relevant	Quite relevant	Highly relevant
Was triage category appropriate based on triage information / observations?	1, Yes<br>2, No	Not relevant	Somewhat relevant	Quite relevant	Highly relevant
If patient deteriorated before medical officer review, was the triage category appropriately upgraded per the ATS?	1, Yes<br>2, No<br>3, N/A - no deterioration before review	Not relevant	Somewhat relevant	Quite relevant	Highly relevant
Date and time of first nurse treatment	date_time	Not relevant	Somewhat relevant	Quite relevant	Highly relevant
Type of nurse treatment initiated	(free text)	Not relevant	Somewhat relevant	Quite relevant	Highly relevant
Date and time of medical officer review	date_time	Not relevant	Somewhat relevant	Quite relevant	Highly relevant
ED Diagnosis	(free text)	Not relevant	Somewhat relevant	Quite relevant	Highly relevant
Time spent in ED (hrs)	(calculated)	Not relevant	Somewhat relevant	Quite relevant	Highly relevant

Do you have any feedback about any of the items in Section 3: ED performance?

Section 4: Communication and care planning

Section 4: Communication and care planning

Rationale: The patient should be an active participant in preparing for EOL. When not possible, a substitute decision maker should be identified and consulted. Existing advance care directives should be identified, and care planning should occur with the patient and their family / carer and communicated to all staff involved in the patient's care.

Question	*Potential responses*	*Relevance*			
Did the patient have a legally appointed decision- maker documented?	1, Yes<br>2, No	Not relevant	Somewhat relevant	Quite relevant	Highly relevant
If yes, was the legally appointed decision maker consulted about health care decisions by the ED clinician?	1, Yes<br>2, No<br>3, Not documented	Not relevant	Somewhat relevant	Quite relevant	Highly relevant
Prior to presentation was there a hospital resuscitation form on file?	1, Yes<br>2, No	Not relevant	Somewhat relevant	Quite relevant	Highly relevant
If yes, is there any evidence the previous resuscitation plan was considered by the ED clinician?	1, Yes, documented in patient notes<br>2, Yes, documented on new resuscitation plan<br>3, No<br>4, Not documented	Not relevant	Somewhat relevant	Quite relevant	Highly relevant

Prior to presentation was there a written advance care plan or advance health directive on file?	1, Yes<br>2, No	Not relevant	Somewhat relevant	Quite relevant	Highly relevant
If yes, is there any evidence the ACD was considered by the ED clinician?	1, Yes, documented in patient notes<br>2, Yes, documented on new resuscitation plan<br>3, No<br>4, Not documented	Not relevant	Somewhat relevant	Quite relevant	Highly relevant
Is there any documentation indicating that the patient's preferences for care were discussed whilst in the ED?	1, Yes<br>2, No	Not relevant	Somewhat relevant	Quite relevant	Highly relevant
At any time was a resuscitation plan documented this presentation?	1, Yes_in the ED<br>2, Yes_in the ICU<br>3, Yes_on the ward<br>4, No resuscitation plan done	Not relevant	Somewhat relevant	Quite relevant	Highly relevant
Date and Time of first resuscitation plan this presentation	date_time	Not relevant	Somewhat relevant	Quite relevant	Highly relevant
Time from presentation to first resus plan	(calculated)	Not relevant	Somewhat relevant	Quite relevant	Highly relevant
	1, No pharyngeal suction<br>2, No supplemental oxygen<br>3, No non-invasive ventilation<br>4, No bag & mask ventilation<br>5, No intubation<br>6, No referral to ICU				

What limitations of treatment were explicitly stated in the documentation of the first resuscitation plan?	7, No CPR<br>8, No other non-urgent interventions (e.g. vascular access, blood products, antibiotics, NG feeds/fluids, imaging, pathology, IV fluids)<br>9, No clinical review call<br>10, No rapid response call<br>11, N/A no resuscitation plan	Not relevant	Somewhat relevant	Quite relevant	Highly relevant
Is there any documentation indicating the patient / family were involved in decision-making about the resuscitation plan?<br><br>*If the patient or family were not involved in the development of the resuscitation plan, is there a reason why not documented?*	1, Yes - patient & family<br>2, Yes -patient only<br>3, Yes - family only<br>4, No<br>5, N/A - no resuscitation plan<br><br>*(Free text)*	Not relevant	Somewhat relevant	Quite relevant	Highly relevant
Was the resuscitation plan revised/changed at any time?	1, Yes<br>2, No	Not relevant	Somewhat relevant	Quite relevant	Highly relevant
Date and Time of first revision to resuscitation plan<br><br>*If the resuscitation plan was changed, please indicate what changes were made*	date_time<br><br>*(free text)*	Not relevant	Somewhat relevant	Quite relevant	Highly relevant
At any point was there evidence or conflicting statements that might create confusion about the patient's resuscitation status or the medical	1, Yes<br>2, No<br>3, N/A - no	Not relevant	Somewhat relevant	Quite relevant	Highly relevant

treatments that were limited?<br><br>*If yes, please describe*	resuscitation plan<br><br>*(free text)*	◯	◯	◯	◯
Was the patient referred to the palliative care team during this presentation?<br><br>*Is yes, date and time of referral*	1, Yes<br><br>2, No<br><br>*date_time*	Not relevant ◯	Somewhat relevant ◯	Quite relevant ◯	Highly relevant ◯
Was the patient's usual GP / GP practice contacted for information regarding patient's usual status, current palliative care arrangements and illness trajectory/likelihood of death?	1, Yes<br><br>2, No<br><br>3, Not documented	Not relevant ◯	Somewhat relevant ◯	Quite relevant ◯	Highly relevant ◯
Was the patient's usual GP / GP practice sent a discharge summary following the patient's death?	1, Yes<br><br>2, No	Not relevant ◯	Somewhat relevant ◯	Quite relevant ◯	Highly relevant ◯

Do you have any feedback about any of the items in Section 4: Communication and care planning?

Section 5: Recognition of dying

Section 5: Recognition of dying

Rationale: To initiate appropriate EOL care at the right time, recognition of dying is important. The integration of the validated CriSTAL tool will determine if patients were at risk of dying on presentation to the ED and whether this was recognised.

Question	*Potential responses*	*Relevance*			
Total CriSTAL score<br>Please refer to the attached pdf for the included data items which are collected to obtain the CriSTAL score	(calculated from pre-collected data fields	Not relevant	Somewhat relevant	Quite relevant	Highly relevant

Attachment: Criteria for Screening and Triaging to Appropriate aLternative care.pdf (142.5 kB)	- please see pdf attached)				
Was the patient at high risk of dying (CriSTAL score >6) on arrival to the ED?	1, Yes<br>2, No	Not relevant	Somewhat relevant	Quite relevant	Highly relevant
Is there documented indication that the patient was actually dying?	1, Yes<br>2, No	Not relevant	Somewhat relevant	Quite relevant	Highly relevant
If yes, date and time<br>*Time of recognition until death (hrs)*	date_time<br>*(calculated)*	Not relevant	Somewhat relevant	Quite relevant	Highly relevant
Is there evidence of communication with the patient and/or family that the patient was dying?	1, Yes - patient & family<br>2, Yes - patient only<br>3, Yes - family only<br>4, No<br>5, N/A - dying not recognised	Not relevant	Somewhat relevant	Quite relevant	Highly relevant
Did the patient have a palliative/comfort care ONLY plan documented at any time during the admission?<br>*If yes, date / time*	1, Yes - in the ED<br>2, Yes - in the ICU<br>3, Yes - on the ward<br>4, No<br>*date_time*	Not relevant	Somewhat relevant	Quite relevant	Highly relevant
If a palliative/comfort care plan was documented was it communicated to the patient and/or family?	1, Yes - patient & family<br>2, Yes - patient only<br>3, Yes - family only<br>4, No	Not relevant	Somewhat relevant	Quite relevant	Highly relevant

Do you have any feedback about any of the items in Section 5: Recognition of dying?

Section 6: Care delivery

Section 6: Care delivery

Rationale: A holistic assessment of needs is an integral component of EOL care delivery. Anticipating and managing distressing symptoms is essential to prevent and relieve suffering. Care should be delivered in an environment conducive to the needs and preferences of the patient.

Question	*Potential responses*	*Relevance*			
Is there documented evidence of an assessment of the following needs :					
Agitation / delirium	1, Yes<br>2, No<br>3, Not documented	Not relevant	Somewhat relevant	Quite relevant	Highly relevant
Anxiety / distress	1, Yes<br>2, No<br>3, Not documented	Not relevant	Somewhat relevant	Quite relevant	Highly relevant
Bladder function	1, Yes<br>2, No<br>3, Not documented	Not relevant	Somewhat relevant	Quite relevant	Highly relevant
Bowel function	1, Yes<br>2, No<br>3, Not documented	Not relevant	Somewhat relevant	Quite relevant	Highly relevant
Dyspnoea / breathing difficulty	1, Yes<br>2, No<br>3, Not documented	Not relevant	Somewhat relevant	Quite relevant	Highly relevant
Emotional / psychological	1, Yes<br>2, No	Not relevant	Somewhat relevant	Quite relevant	Highly relevant

	3, Not documented				
Eye / mouth care	1, Yes<br>2, No<br>3, Not documented	Not relevant	Somewhat relevant	Quite relevant	Highly relevant
Nausea / vomiting	1, Yes<br>2, No<br>3, Not documented	Not relevant	Somewhat relevant	Quite relevant	Highly relevant
Noisy breathing / death rattle / excess secretions	1, Yes<br>2, No<br>3, Not documented	Not relevant	Somewhat relevant	Quite relevant	Highly relevant
Nutrition / hydration	1, Yes<br>2, No<br>3, Not documented	Not relevant	Somewhat relevant	Quite relevant	Highly relevant
Pain	1, Yes<br>2, No<br>3, Not documented	Not relevant	Somewhat relevant	Quite relevant	Highly relevant
Social / practical	1, Yes<br>2, No<br>3, Not documented	Not relevant	Somewhat relevant	Quite relevant	Highly relevant
Spiritual / religious / cultural	1, Yes<br>2, No<br>3, Not documented	Not relevant	Somewhat relevant	Quite relevant	Highly relevant
Pastoral care	1, Yes<br>2, No<br>3, Not documented	Not relevant	Somewhat relevant	Quite relevant	Highly relevant
Is there documented evidence that anticipatory medication was prescribed appropriately for symptoms likely to occur in the last days of life? (must include one	1, Yes<br>2, No	Not relevant	Somewhat relevant	Quite relevant	Highly relevant

opioid, one sedative and one antisecretory)					
If yes, date and time of prescription<br>*Time from prescription to patient death*	date_time<br>*Calculated*	Not relevant	Somewhat relevant	Quite relevant	Highly relevant
If anticipatory medications (opioid, sedative and antisecretory) were not prescribed appropriately, please provide details	(free text)	Not relevant	Somewhat relevant	Quite relevant	Highly relevant
Once a decision for EOL care was made were regular medications which may have been thought to be unnecessary ceased?	1, Yes<br>2, No	Not relevant	Somewhat relevant	Quite relevant	Highly relevant
Was there use of a continuous subcutaneous syringe driver if required?	1, Yes<br>2, No<br>3, N/A - syringe driver not required	Not relevant	Somewhat relevant	Quite relevant	Highly relevant
Once a decision for EOL care was made were routine care processes which may have been thought to be unnecessary ceased? (e.g. routine observations, blood tests, IV fluids)	1, Yes<br>2, No<br>3, N/A	Not relevant	Somewhat relevant	Quite relevant	Highly relevant
If routine care processes were not ceased once a decision for EOL was made, please provide details	(free text)	Not relevant	Somewhat relevant	Quite relevant	Highly relevant
Did the patient receive any of the following interventions in their last 48 hours of life?					
Cardiopulmonary resuscitation	1, Yes<br>2, No	Not relevant	Somewhat relevant	Quite relevant	Highly relevant
Intubation / mechanical ventilation	1, Yes<br>2, No	Not relevant	Somewhat relevant	Quite relevant	Highly relevant
Non-invasive ventilation	1, Yes<br>2, No	Not relevant	Somewhat relevant	Quite relevant	Highly relevant
Chemotherapy	1, Yes	Not relevant	Somewhat relevant	Quite relevant	Highly relevant

	2, No	◯	◯	◯	◯
Dialysis	1, Yes<br>2, No	Not relevant ◯	Somewhat relevant ◯	Quite relevant ◯	Highly relevant ◯
Blood tests	1, Yes<br>2, No	Not relevant ◯	Somewhat relevant ◯	Quite relevant ◯	Highly relevant ◯
Medical imaging<br>*Specify*	1, Yes<br>2, No<br>*Xray|CT scan|*<br>*MRI|Ultrasound|PET scan*	Not relevant ◯	Somewhat relevant ◯	Quite relevant ◯	Highly relevant ◯
Artificial nutrition	1, Yes<br>2, No	Not relevant ◯	Somewhat relevant ◯	Quite relevant ◯	Highly relevant ◯
Artificial hydration	1, Yes<br>2, No	Not relevant ◯	Somewhat relevant ◯	Quite relevant ◯	Highly relevant ◯
Is there any evidence that interventions were performed against documented wishes/ ACD or resuscitation plan?<br>*If yes, please detail*	1, Yes<br>2, No<br>3, N/A no resuscitation plan / ACD or documented wishes	Not relevant ◯	Somewhat relevant ◯	Quite relevant ◯	Highly relevant ◯
Did the patient experience any MET calls either in the ED or after they were transferred from the ED?	1, Yes<br>2, No<br>3, N/A - per resus plan patient is not for MET calls or ICU	Not relevant ◯	Somewhat relevant ◯	Quite relevant ◯	Highly relevant ◯
If yes, how many?	1, 1-2<br>2, 3-4<br>3, 5+	Not relevant ◯	Somewhat relevant ◯	Quite relevant ◯	Highly relevant ◯
Date / time of first MET call	date_time	Not relevant ◯	Somewhat relevant ◯	Quite relevant ◯	Highly relevant ◯
Was a palliative / comfort only plan	1, Yes	Not relevant	Somewhat relevant	Quite relevant	Highly relevant

initiated as a result of a MET call?	2, No	○	○	○	○
Was the patient in a single room at the time of death?	1, Yes<br>2, No<br>3, Unsure	Not relevant ○	Somewhat relevant ○	Quite relevant ○	Highly relevant ○
Is there evidence that the patients preferred place of death was documented?	1, Yes<br>2, No	Not relevant ○	Somewhat relevant ○	Quite relevant ○	Highly relevant ○
Were attempts at terminal discharge made, if this was in line with patient wishes?<br>*If no, or if attempts at terminal discharge were unsuccessful, is there a reason why documented? Please provide details*	1, Yes<br>2, No<br>3, N/A - patients preferred place of death was hospital<br>*(Free text)*	Not relevant ○	Somewhat relevant ○	Quite relevant ○	Highly relevant ○

Do you have any feedback about any of the items in Section 6: Care delivery

Is there documented evidence of an assessment of the following needs of families / carers:

Section 7: Needs of families / carers

Rationale: Engaging with and supporting families / carers, including bereavement care after the death of the patient, is reflective of good EOL practices.

Question	*Potential responses*	*Relevance*			
Were family / carers present at time of death?	1, Yes<br>2, No	Not relevant	Somewhat relevant	Quite relevant	Highly relevant

	3, Unsure	○	○	○	○
If no, is there evidence they were contacted / offered to be present	1, Yes - family declined<br>2, No<br>3, Other (details)	Not relevant ○	Somewhat relevant ○	Quite relevant ○	Highly relevant ○
Is there documented evidence of an assessment of the following needs of families / carers?					
Emotional / psychological	1, Yes<br>2, No<br>3, Not documented<br>4, Family not present	Not relevant ○	Somewhat relevant ○	Quite relevant ○	Highly relevant ○
Spiritual / religious / cultural	1, Yes<br>2, No<br>3, Not documented<br>4, Family not present	Not relevant ○	Somewhat relevant ○	Quite relevant ○	Highly relevant ○
Practical	1, Yes<br>2, No<br>3, Not documented<br>4, Family not present	Not relevant ○	Somewhat relevant ○	Quite relevant ○	Highly relevant ○
Is there evidence that social work was offered?	1, Yes - social work involved<br>2, Yes - family or patient declined social work<br>3, Yes - referred but not reviewed<br>4, No<br>5, Not documented	Not relevant ○	Somewhat relevant ○	Quite relevant ○	Highly relevant ○
	1, Yes				

Is there evidence the family were given information on procedures/tasks after death?	2, No<br>3, Not documented	Not relevant ○	Somewhat relevant ○	Quite relevant ○	Highly relevant ○
Is there evidence that families were provided with written bereavement information?	1, Yes<br>2, No<br>3, Not documented	Not relevant ○	Somewhat relevant ○	Quite relevant ○	Highly relevant ○

Do you have any feedback about any of the items in Section 7: Needs of families / carers

Submit

Save & Return Later
