# Supplementary material for: Development of an Audit Tool to Evaluate End of Life Care in the Emergency Department: A Face and Content Validity Study
Source: J Eval Clin Pract. 2025 Feb 19;31(1):e70041. doi: 10.1111/jep.70041 (PMC11839938; doi:10.1111/jep.70041)
Supplement: Supplementary file 3 — Supporting information. [file JEP-31-0-s001.pdf]

Item and Scale content validity results of the Emergency Department End of Life Care audit tool

CVI	CVI round one		CVI round two	
**Item**	**I-CVI**	**Outcome**	**I-CVI**	**Outcome**
**Section 1: Patient characteristics**				
Basic demographics<br><br>(Age, sex, country of birth, religion, indigenous status, place of usual residence)	0.91	Included	-	-
Was the patient known to have a terminal / life-limiting illness (e.g., Cancer, COPD, heart failure dementia)	1.00	Included	-	-
How many times was the patient admitted to an acute hospital in the 12 months prior to this hospital admission?	0.91	Included	-	-
Was the patient known to community palliative care services?	0.64	Deleted	-	-
**Section 2: Circumstances of death**				
Cause of death	1.00	Included		
Speciality with overall responsibility for the patients care at time of death	0.64	Needs Revision	0.80	Included
Specific ward / area patient died	0.55	Needs Revision	0.78	Included
Was the patient admitted to the ICU at any time during this admission?	0.82	Included	-	-
**Section 3: ED Performance**				
Mode of arrival	0.64	Deleted	-	-
Presenting complaint	0.82	Included	-	-
Triage category	0.82	Included	-	-
Triage information	0.55	Deleted	-	-
Was triage category appropriate based on triage information / observations?	0.64	Needs Revision	0.75	Revised
If patient deteriorated before medical officer review, was the triage category appropriately upgraded per the ATS?	0.73	Needs Revision	0.78	Included
Date and time of first nurse<br><br>treatment	1.00	Included	-	-
Type of nurse treatment initiated	0.82	Included	-	-

Date and time of medical officer review	1.00	Included	-	-
ED Diagnosis	0.91	Included	-	-
Time spent in ED (hrs)	1.00	Included	-	-
**Section 4: Communication and care planning**				
Did the patient have a legally appointed decision- maker documented?	0.82	Included	-	-
If yes, was the legally appointed decision maker consulted about health care decisions by the ED clinician?	1.00	Included	-	-
Prior to presentation was there a hospital resuscitation form on file?	0.73	Needs Revision	0.80	Included
If yes, is there any evidence the previous resuscitation plan was considered by the ED clinician?	0.90	Included	-	-
Prior to presentation was there a written advance care plan or advance health directive on file?	1.00	Included	-	-
If yes, is there any evidence the ACD was considered by the ED clinician?	1.00	Included	-	-
Is there any documentation indicating that the patient's preferences for care were discussed whilst in the ED?	1.00	Included	-	-
At any time was a resuscitation plan documented this presentation?	1.00	Included	-	-
Date and Time of first resuscitation plan this presentation	0.82	Included	-	-
Time from presentation to first resus plan	0.73	Needs Revision	0.70	Deleted
What limitations of treatment were explicitly stated in the documentation of the first resuscitation plan?	0.82	Included	-	-
Is there any documentation indicating the patient / family were involved in decision-making about the resuscitation plan?<br>*If the patient or family were not involved in the development of the resuscitation plan, is there a reason why not documented?*	1.00	Included	-	-
Was the resuscitation plan revised/changed at any time?	0.73	Needs Revision	0.80	Included

Date and Time of first revision to resuscitation plan<br><br>*If the resuscitation plan was changed, please indicate what changes were made*	0.64	Deleted	-	-
At any point was there evidence or conflicting statements that might create confusion about the patient's resuscitation status or the medical treatments that were limited?<br><br>*If yes, please describe*	0.73	Needs Revision	0.80	Included
Was the patient referred to the palliative care team during this presentation?<br><br>*If yes, date and time of referral*	0.82	Included	-	-
Was the patient's usual GP / GP practice contacted for information regarding patient's usual status, current palliative care arrangements and illness trajectory/likelihood of death?	0.55	Deleted	-	-
Was the patient's usual GP / GP practice sent a discharge summary following the patient's death?	0.64	Deleted	-	-
**Section 5: Recognition of dying**				
Was the patient at high risk of dying (CriSTAL score >6) on arrival to the ED?	0.73	Needs Revision	0.90	Included
Is there documented indication that the patient was actually dying?	0.82	Included	-	-
If yes, date and time<br><br>*Time of recognition until death (hrs)*	0.73	Needs Revision	0.80	Included
Is there evidence of communication with the patient and/or family that the patient was dying?	1.00	Included	-	-
Did the patient have a palliative/comfort care ONLY plan documented at any time during the admission?<br><br>*If yes, date / time*	1.00	Included	-	-
If a palliative/comfort care plan was documented, was it communicated to the patient and/or family?	1.00	Included	-	-
**Section 6: Care delivery**				
Is there documented assessment of the following:				
Agitation / delirium	1.00	Included	-	-

Anxiety / distress	0.91	Included	-	-
Bladder function	0.91	Included	-	-
Bowel function	0.82	Included	-	-
Dyspnoea / breathing difficulty	1.00	Included	-	-
Emotional / psychological	0.91	Included	-	-
Eye / mouth care	0.82	Included	-	-
Nausea / vomiting	0.73	Needs Revision	0.60	Deleted
Noisy breathing / death rattle / excess secretions	0.91	Included	-	-
Nutrition / hydration	0.91	Included	-	-
Pain	1.00	Included	-	-
Social / practical	0.73	Needs Revision	0.90	Included
Spiritual religious / cultural	1.00	Included	-	-
Pastoral care	0.82	Included	-	-
Did the patient receive any of the following interventions in their last 48 hours of life?				
Cardiopulmonary resuscitation	0.82	Included	-	-
Intubation / mechanical ventilation	0.91	Included	-	-
Non-invasive ventilation	0.91	Included	-	-
Chemotherapy	0.91	Included	-	-
Dialysis	0.91	Included	-	-
Blood tests	0.82	Included	-	-
Medical imaging<br>*Specify*	0.82	Included	-	-
Artificial nutrition	0.91	Included	-	-
Artificial hydration	0.82	Included	-	-
Is there documented evidence that anticipatory medication was prescribed appropriately for symptoms likely to occur in the last days of life? (must include one opioid, one sedative and one antisecretory)	1.00	Included	-	-
If yes, date and time of prescription<br>*Time from prescription to patient death*	0.73	Needs Revision	0.80	Included

If anticipatory medications (opioid, sedative and antisecretory) were not prescribed appropriately, please provide details	0.82	Included	-	-
Once a decision for EOL care was made were regular medications which may have been thought to be unnecessary ceased?	0.82	Included	-	-
Was there use of a continuous subcutaneous syringe driver if required?	0.91	Included	-	-
Once a decision for EOL care was made were routine care processes which may have been thought to be unnecessary ceased? (e.g. routine observations, blood tests, IV fluids)	0.82	Included	-	-
If routine care processes were not ceased once a decision for EOL was made, please provide details	0.73	Needs Revision	0.70	Revised
Is there any evidence that interventions were performed against documented wishes/ ACD or resuscitation plan?<br>*If yes, please detail*	1.00	Included	-	-
Did the patient experience any MET calls either in the ED or after they were transferred from the ED?	1.00	Included	-	-
If yes, how many?	0.73	Needs Revision	1.00	Included
Date / time of first MET call	0.45	Deleted	-	-
Was a palliative / comfort only plan initiated as a result of a MET call?	0.91	Included	-	-
Was the patient in a single room at the time of death?	0.91	Included	-	-
Is there evidence that the patients preferred place of death was documented?	1.00	Included	-	-
Were attempts at terminal discharge made if this was in line with patient wishes?<br>*If no, or if attempts at terminal discharge were unsuccessful, is there a reason why documented? Please provide details*	0.82	Included	-	-
**Section 7: Needs of families and carers**				
Were family / carers present at time of death?	0.82	Included	-	-
If no, is there evidence they were contacted / offered to be present	0.91	Included	-	-

Is there evidence that social work was offered?	0.91	Included	-	-
Is there evidence the family were given information on procedures/tasks after death?	0.91	Included	-	-
Is there evidence that families were provided with written bereavement information?	0.55	Needs Revision	0.80	Included
Is there documented evidence of an assessment of the following needs of families / carers:				
Emotional / psychological	0.91	Included	-	-
Spiritual / religious / cultural	0.90	Included	-	-
Practical	0.91	Included	-	-
**Questions added following first round:**				
Is there evidence that bereavement risk screening was undertaken?	-	-	0.50	Deleted
Were specialist Palliative Care contacted for advice?	-	-	0.80	Included
			**S-CVI/Ave**	
			0.90	Excellent

Key: ACD – Advance Care Directive, ATS – Australasian Triage Scale, COPD – Chronic Obstructive Pulmonary Disease, CriSTAL – Criteria for Screening and Triaging to Appropriate aLternative care, CVI – Content Validity Index, ED - Emergency Department, EOL – End of Life, GP – General Practitioner, ICU - Intensive Care Unit, I-CVI – Item Content Validity Index, IV – intravenous, MET – Medical Emergency Team, S-CVI – Scale Content Validity Index
