# Supplementary material for: Development of an Audit Tool to Evaluate End of Life Care in the Emergency Department: A Face and Content Validity Study
Source: J Eval Clin Pract. 2025 Feb 19;31(1):e70041. doi: 10.1111/jep.70041 (PMC11839938; doi:10.1111/jep.70041)
Supplement: Supplementary file 4 — Supporting information. [file JEP-31-0-s003.pdf]

Emergency Department End of Life Care audit tool items following face and content validity

**Field Label**	**Field Attributes**
Record ID	Text
Audit category for patient	1 Patient died in ED<br>2 Patient died in ICU, within 48hours<br>3 Patient died on inpatient ward, within 48hours
Section 1: Patient characteristics	
Medical Record Number	Free text
First name	Free text
Last name	Free text
Date of Birth	Free text (date, d/m/y)
Age	Calculated
Sex	1 Female<br>2 Male
Country of Birth	Free text
Religion	1 No religion<br>2 Christian (including Church of England, Catholic, Protestant, Presbyterian, and all other Christian denominations)<br>3 Buddhist<br>9 Islam<br>4 Hindu<br>5 Jewish<br>6 Muslim<br>7 Other (Specify – free text)<br>8 Not recorded
Indigenous Status	1 Aboriginal but not Torres Strait Islander origin<br>2 Torres Strait Islander but not Aboriginal origin<br>3 Both Aboriginal and Torres Strait Islander origin<br>4 Neither Aboriginal nor Torres Strait Islander origin<br>5 Not recorded
Place of usual residence	1 Home<br>2 Residential Aged Care Facility<br>3 Group Home<br>4 Other (Specify - free text)
Was the patient known to have a terminal / life-limiting illness (e.g. Cancer, COPD, heart failure, dementia)? If yes, please specify	1 Yes (Specify – free text)<br>2 No
How many times was the patient admitted to an acute hospital in the 12 months prior to this hospital admission?	1 None<br>2 1-2 times<br>3 3-5 times<br>4 More than 5 times<br>5 Not known
Previous ICU admission for at least one night in past year	1 Yes<br>2 No<br>3 Not documented
Do you have any further comments regarding Section 1: Patient characteristics?	Free text
Section 2: Circumstances of death	
Date and Time of Death	Free text (date d/m/y)
Cause of death	Free text
Other risk factors / predictors (Tick as many as relevant)<br>*CriSTAL	1 Advanced malignancy<br>2 Chronic kidney disease<br>3 Chronic heart failure<br>4 Chronic obstructive pulmonary disease<br>5 New cerebrovascular disease<br>6 New myocardial infarction

	7	Moderate/severe liver disease
Evidence of cognitive impairment, any of the following;<br>Dementia / Long term mental disorder / Behavioural Alterations / Mental disability from stroke<br>*CriSTAL	1<br>2	Yes<br>No
History of pneumonia in past 6 months?<br>*CriSTAL	1<br>2	Yes<br>No
Polypharmacy, defined as 7 or more current prescription medications?<br>*CriSTAL	1<br>2	Yes<br>No
Fall or fracture in last 6 months?<br>*CriSTAL	1<br>2	Yes<br>No
Nutritional vulnerability on admission<br>(Any of the following; Malnutrition / History of unintentional weight loss / Sarcopenia / Feeding dependency / Feeding tube / Modified diet types)<br>*CriSTAL	1<br>2	Yes<br>No
Evidence of frailty (Clinical Frailty Score)<br>*CriSTAL	1<br>2<br>3<br>4<br>5<br>6<br>7<br>8<br>9	1. Very fit<br>2. Well<br>3. Managing well<br>4. Vulnerable<br>5. Mildly frail<br>6. Moderately frail<br>7. Severely frail<br>8. Very severely frail<br>9. Terminally ill
Speciality with overall responsibility for the patients care at time of death	1<br>2<br>3<br>4<br>5<br>6<br>7<br>8<br>9<br>10<br>11<br>12<br>13<br>14<br>15<br>16<br>17<br>18<br>19<br>20<br>21<br>22	Emergency<br>Cardiology<br>Endocrinology<br>Ear Nose and Throat<br>Gastroenterology<br>General Medicine<br>General surgery<br>Geriatrics<br>Gynaecology<br>Haematology<br>Infectious diseases<br>Neurology<br>Neurosurgery<br>Obstetrics<br>Orthopaedics<br>Paediatrics<br>Palliative Care<br>Rehabilitation<br>Respiratory Medicine<br>Urology<br>Vascular<br>Other (free text)
Specific ward / area patient died	Free text	
Was the patient admitted to the ICU at anytime during this admission?	1<br>2	Yes<br>No
If the patient was admitted to the ICU, time spent in ICU (hrs)	Free text	
Total hospital length of stay (hrs)	Calculation	

Do you have any further comments regarding Section 2: Circumstances of death?	Free text
Section3: ED performance	
Date and time of triage	Free text (date – d/m/y)
Presenting complaint	Free text
Triage category	1 1<br>2 2<br>3 3<br>4 4<br>5 5
Was triage category appropriate based on triage information / observations?	1 Yes<br>2 No
If triage category not appropriate, please provide details	Free text
If patient deteriorated before medical officer review, was the triage category appropriately upgraded per the ATS?	1 Yes<br>2 No<br>3 N/A - no deterioration before review
If triage category was not appropriately upgraded if patient deteriorated before medical officer review, please provide details	Free text
Deterioration criteria on presentation (please select all that are applicable)<br>*CriSTAL	1 Decreased LOC: GCS change >2 or AVPU = P or U<br>2 Systolic blood pressure < 90mmHg<br>3 Respiratory rate < 5 or >30 per minute<br>4 Pulse rate < 40 or >140 per minute<br>5 Need for oxygen therapy or known oxygen saturation < 90%<br>6 Hypoglycaemia: BGL 1.0 - 4.0 mmol/L<br>7 Repeat or prolonged seizures (>5 minutes duration or >= 2 per day)<br>8 Low urinary output (< 15 ml/hour or < 0.5 ml/kg/hour)
Meets >= 2 deterioration criteria on presentation *CriSTAL	1 Yes<br>2 No
Date and time of first nurse treatment	Free text (date - d/m/y)
Type of nurse treatment initiated	Free text
Date and time of medical officer review	Free text (date - d/m/y)
Date and time depart ready	Free text (date - d/m/y)
ED Diagnosis	Free text
Date and time of transfer to ward, if left ED	Free text (date - d/m/y)
Time spent in ED (hrs) (Audit category = died on ward/ICU)	Calculation
Time spent in ED (hrs) (Audit category = died in ED)	Calculation
Do you have any further comments regarding section 3: ED performance?	Free text
Section 4: Communication and care planning	
Did the patient have a legally appointed decision-maker documented?	1 Yes<br>2 No
If yes, was the legally appointed decision maker consulted about health care decisions by the ED clinician?	1 Yes<br>2 No<br>3 Not documented
Prior to presentation was there a previous hospital resuscitation form on file?	1 Yes<br>2 No
If yes, is there any evidence the previous resuscitation plan was considered by the ED clinician?	1 Yes, documented in patient notes<br>2 Yes, documented on new resuscitation plan<br>3 No<br>4 Not documented
Prior to presentation was there a written advance care plan or advance health directive on file?	1 Yes<br>2 No

If yes, is there any evidence the ACD was considered by the ED clinician?	1 Yes, documented in patient notes<br>2 Yes, documented on new resuscitation plan<br>3 No<br>4 Not documented
Is there any documentation indicating that the patient's preferences for care were discussed whilst in the ED?	1 Yes<br>2 No
At any time was a resuscitation plan documented?	1 Yes - in the ED<br>2 Yes - in the ICU<br>3 Yes - on the ward<br>4 No resuscitation plan done
Date and Time of first resuscitation plan	Free text (date - d/m/y)
What limitations of treatment were explicitly stated in the documentation of the first resuscitation plan?	1 No pharyngeal suction<br>2 No supplemental oxygen<br>3 No non-invasive ventilation<br>4 No bag & mask ventilation<br>5 No intubation<br>6 No referral to ICU<br>7 No CPR<br>8 No other non-urgent interventions (e.g., vascular access, blood products, antibiotics, NG feeds/fluids, imaging, pathology, IV fluids)<br>9 No clinical review call<br>10 No rapid response call<br>11 N/A no resuscitation plan
If other non-urgent interventions were limited, please detail here	Free text
Is there any documentation indicating the patient / family were involved in decision-making about the resuscitation plan?	1 Yes - patient & family<br>2 Yes -patient only<br>3 Yes - family only<br>4 No<br>5 N/A - no resuscitation plan
If the patient or family were not involved in the development of the resuscitation plan, is there a reason why not documented?	Free text
Was the resuscitation plan revised/changed at any time?	1 Yes<br>2 No
If the resuscitation plan was changed, please indicate what changes were made	Free text
At any point was their evidence or conflicting statements that might create confusion about the patient's resuscitation status or the medical treatments that were limited?	1 Yes<br>2 No<br>3 N/A - no resuscitation plan
If yes, please describe	Free text
Was the patient referred to the palliative care team during this presentation?	1 Yes<br>2 No
If yes, date and time of referral	Free text (date - d/m/y)
Time between palliative care referral and death	Calculation
If no, were specialist palliative care contacted for advice?	1 Yes<br>2 No
Do you have any further comments regarding section 4: Communication and care planning	Free text

CriSTAL Tool	
Age >65<br>Admitted via the ED<br>Nursing home resident / Supportive accommodation<br>Previous hospitalisation score<br>Previous ICU admission score<br>Deterioration score<br>Risk 1 score<br>Risk 2 score<br>Risk 3 score<br>Risk 4 score<br>Risk 5 score<br>Risk 6 score<br>Risk 7 score<br>Total score of other risk factors<br>Cognitive impairment score<br>Pneumonia score<br>Polypharmacy score<br>Fall or fracture score<br>Nutrition score<br>Frailty score<br>Total CriSTAL score	Calculation
Section 5: Recognition of dying	
Was the patient at high risk of dying (CriSTAL score >6) on arrival to the ED?	1 Yes<br>2 No
Is there documented indication that the patient was actually dying?	1 Yes<br>2 No
If yes, date and time	Free text (date - d/m/y)
Time of recognition until death (hrs)	Calculation
Is there evidence of communication with the patient and/or family that the patient was dying?	1 Yes - patient & family<br>2 Yes - patient only<br>3 Yes - family only<br>4 No<br>5 N/A - dying not recognised
Did the patient have a palliative/comfort care ONLY plan documented at any time during the admission?	1 Yes - in the ED<br>2 Yes - in the ICU<br>3 Yes - on the ward<br>4 No
If yes, date / time	Free text (date - d/m/y)
Time from comfort plan to time of death	Calculated
If a palliative/comfort care plan was documented, was it communicated to the patient and/or family?	1 Yes - patient & family<br>2 Yes - patient only<br>3 Yes - family only<br>4 No
Any further comments regarding Section 5: Recognition of dying?	Free text
Section 6: Care delivery	
Is there documented evidence of an assessment of the following needs:<br>Agitation / delirium<br>Anxiety / distress<br>Bladder function<br>Bowel function<br>Dyspnoea / breathing difficulty<br>Emotional / psychological	1 Yes<br>2 No<br>3 Not documented

Eye / mouth care<br>Noisy breathing / death rattle / excess secretions<br>Nutrition / hydration<br>Pain<br>Social<br>Spiritual / religious / cultural<br>Pastoral care	
Please add any additional details regarding care needs assessment	Free text
Is there documented evidence that anticipatory medication was prescribed appropriately for symptoms likely to occur in the last days of life? (must include one opioid, one sedative and one antisecretory - of subcutaneous administration)	1 Yes<br>2 No
If yes, date and time of prescription	Free text (date - d/m/y)
Time from prescription of anticipatory medications to death	Calculation
If anticipatory medications (opioid, sedative and antisecretory) were not prescribed appropriately, please provide details	Free text
Once a decision for EOL care was made were regular medications which may have been thought to be unnecessary ceased?	1 Yes<br>2 No<br>3 N/A
Was there use of a continuous subcutaneous syringe driver if required?	1 Yes<br>2 No<br>3 N/A
Once a decision for EOL care was made were routine care processes which may have been thought to be unnecessary ceased? (e.g. routine observations, blood tests, IV fluids)	1 Yes<br>2 No<br>3 N/A
If routine care processes were not ceased once a decision for EOL was made, please provide details	Free text
Did the patient receive any of the following interventions in their last 48hrs of life?<br>Cardiopulmonary resuscitation<br>Intubation / mechanical ventilation<br>Non-invasive ventilation<br>Chemotherapy<br>Dialysis<br>Blood tests<br>Medical imaging<br>Artificial nutrition<br>Artificial hydration	1 Yes<br>2 No
If patient received medical imaging in the last 48hrs of life, please indicate which imaging they received	1 Xray<br>2 CT scan<br>3 MRI<br>4 Ultrasound<br>5 PET scan
Please add any additional details regarding interventions received in the last 48hours of life	Free text
Is there any evidence that interventions were performed against documented wishes/ ACD or resuscitation plan?	1 Yes<br>2 No<br>3 N/A no resuscitation plan / ACD or documented wishes
If yes, please detail	Free text
Did the patient experience any MET calls either in the ED or after they were transferred from the ED?	1 Yes<br>2 No

	3 N/A - per resuscitation plan patient is not for MET calls<br>4 N/A - no resuscitation plan
If yes, how many?	1 1-2<br>2 3-4<br>3 5+
Was a palliative / comfort only plan initiated as a result of a MET call?	1 Yes<br>2 No
Was the patient in a single room at the time of death?	1 Yes<br>2 No<br>3 Unsure
Is there evidence that the patients preferred place of death was documented?	1 Yes<br>2 No
Were attempts at terminal discharge made if this was in line with patient wishes?	1 Yes<br>2 No<br>3 N/A - patients preferred place of death was hospital
If no, or if attempts at terminal discharge were unsuccessful, is there a reason why documented? Please provide details	Free text
Any further comments regarding Section 6: Care delivery	Free text
Section 7: Needs of families and carers	
Were family / carers present at time of death?	1 Yes<br>2 No<br>3 Unsure
If no, is there evidence they were contacted / offered to be present?	1 Yes - family declined<br>2 No<br>3 Other (Specify – free text)
Is there documented evidence of an assessment of the following needs of families/carers:<br>Emotional / psychological<br>Spiritual / religious / cultural<br>Practical	1 Yes<br>2 No<br>3 Not documented<br>4 Family not present
Is there evidence that social work was offered?	1 Yes - social work involved<br>2 Yes - family or patient declined social work<br>3 Yes - referred but not reviewed<br>4 No<br>5 Not documented
Is there evidence the family were given information on procedures/tasks after death?	1 Yes<br>2 No<br>3 Not documented
Is there evidence that families were provided with bereavement information?	1 Yes<br>2 No<br>3 Not documented
Any further comments regarding Section 7: Needs of families and carers?	Free text

*CriSTAL: Questions make up part of the total CriSTAL score

Key: ACD – Advance Care Directive, ATS – Australasian Triage Scale, AVPU – Alert, Verbal, Pain, Unconscious, BGL – Blood Glucose Level, COPD – Chronic Obstructive Pulmonary Disease, CT - computerized tomography, CPR – Cardiopulmonary Resuscitation, CriSTAL –Criteria for Screening and Triaging to Appropriate aLternative care, ED - Emergency Department, EOL – End of Life, GCS – Glasgow Coma Scale, ICU - Intensive Care Unit, IV – intravenous, LOC – Level of Consciousness, ml – milliliters, mmHg – millimeters of mercury, MET – Medical Emergency Team, MRI - Magnetic resonance imaging, NG – Nasogastric, PET - positron emission tomography
